# Banding with lesser trochanter fragment using nonabsorbable tape in trochanteric femoral fractures

**DOI:** 10.1051/sicotj/2021032

**Published:** 2021-05-19

**Authors:** Takuya Usami, Naoya Takada, Kazuki Nishida, Hiroaki Sakai, Hidetoshi Iwata, Isato Sekiya, Yoshino Ueki, Hideki Murakami, Gen Kuroyanagi

**Affiliations:** 1 Department of Orthopaedic Surgery, Nagoya City University Graduate School of Medical Sciences Nagoya 467-8601 Aichi Japan; 2 Department of Orthopaedic Surgery, Kainan Hospital Yatomi 498-8502 Aichi Japan; 3 Center for Advanced Medicine and Clinical Research Nagoya University Hospital Nagoya 466-8560 Aichi Japan; 4 Department of Rehabilitation Medicine, Nagoya City University Graduate School of Medical Sciences Nagoya 467-8601 Aichi Japan

**Keywords:** Banding, Lesser trochanter fragment, Nonabsorbable tape, Trochanteric femoral fractures

## Abstract

*Introduction*: Trochanteric femoral fracture is one of the most common fractures in the elderly. Trochanteric femoral fracture with involvement of the lesser trochanter is considered unstable and recognized as having a poor prognosis. However, fixation of lesser trochanter fragment is scarce because of technical difficulties. In this study, we reported the simple surgical procedure and the effect of using nonabsorbable tape in lesser trochanter fixation. *Methods*: From January 2014 to December 2017, 114 patients treated with proximal intramedullary nailing for trochanteric fractures with the lesser trochanter fragment were reviewed. Among patients enrolled in this study, 73 were followed up until radiographic bone union, of which 26 were treated with lesser trochanter fragment banding (group B) and 47 without banding (group N). Radiographs and/or computed tomography images were used to evaluate bone union of the lesser trochanter fragment at three months postoperatively. *Results*: The bone union of the lesser trochanter fragment was achieved in 24 cases (92%) in group B and 30 cases (64%) in group N. Compared with group N, group B showed a significantly increased number of mild and moderate deformities but decreased number of severe deformity and nonunion (*P* < 0.001). Postoperative complications were not observed in both groups. *Conclusions*: From the viewpoint of increasing lesser trochanteric bone union ratio, fixation of the lesser trochanter fragment using nonabsorbable tape in the treatment of trochanteric fractures could be an effective procedure.

## Introduction

More than 1.5 million hip fractures occur worldwide every year [1]. Among hip fractures, trochanteric femoral fractures are common and occur in elderly individuals with osteoporosis. The main reason for this fracture is direct fall [2]. Because of the increased number of elderly individuals in the aging society, the number of trochanteric fractures continues to increase every year [3]. The mortality after hip fracture is reportedly 14% in 6 months and 25% in 1 year if the patients do not undergo surgery [4–6]. Closed reduction and internal fixation using a proximal intramedullary nail is a common surgical procedure for trochanteric fractures [7–9]. Generally, good clinical results using intramedullary nails have been reported. However, some fractures without medial cortical support, including lesser trochanteric fractures, are considered unstable; hence, their clinical results after the surgery may be poor.

The lesser trochanter is a small conical projection located medially inferior to the upper part of the femur. The iliopsoas muscle, the largest muscle in humans, is attached to the lesser trochanter, has an important role in flexion and rotation of the hip joint, and is also known as a postural stabilizer [10].

Trochanteric fractures with lesser trochanter fragments are classified into AO Foundation/Orthopedic Trauma Association (AO/OTA) 31-A1.1, 1.3, A2, and A3.3 [11]. The severely displaced lesser trochanter increases postoperative complications and pain after the treatment of unstable trochanteric fractures [12]. However, because most trochanteric fractures with this fragment are comminuted, anatomical fixation of the lesser trochanter fragment remains challenging. Although it is preferable to fix it in its original position, most orthopedic surgeons do not fix the lesser trochanter fragment because the procedure is difficult. In contrast, Kim et al. reported a surgical method called the *modified candy package technique* to hold the lesser trochanter using twisted steel wires [13]. Although their study obtained good clinical results, we believe that the operative technique is a little complicated and are worried regarding the complications of using metallic materials. We hypothesized that proximal femoral nailing and fixation of the lesser trochanter fragment with nonabsorbable tape increases the union ratio of the lesser trochanter but decreases its deformity and that this surgical procedure does not affect postoperative complications. This study aimed to investigate the bone union ratio of the lesser trochanter fragment, lesser trochanteric deformity, and postoperative complications after the treatment of trochanteric fracture with the lesser trochanter fragment using a proximal intramedullary nail and nonabsorbable tape.

## Methods

### Study design

**Figure 1:**
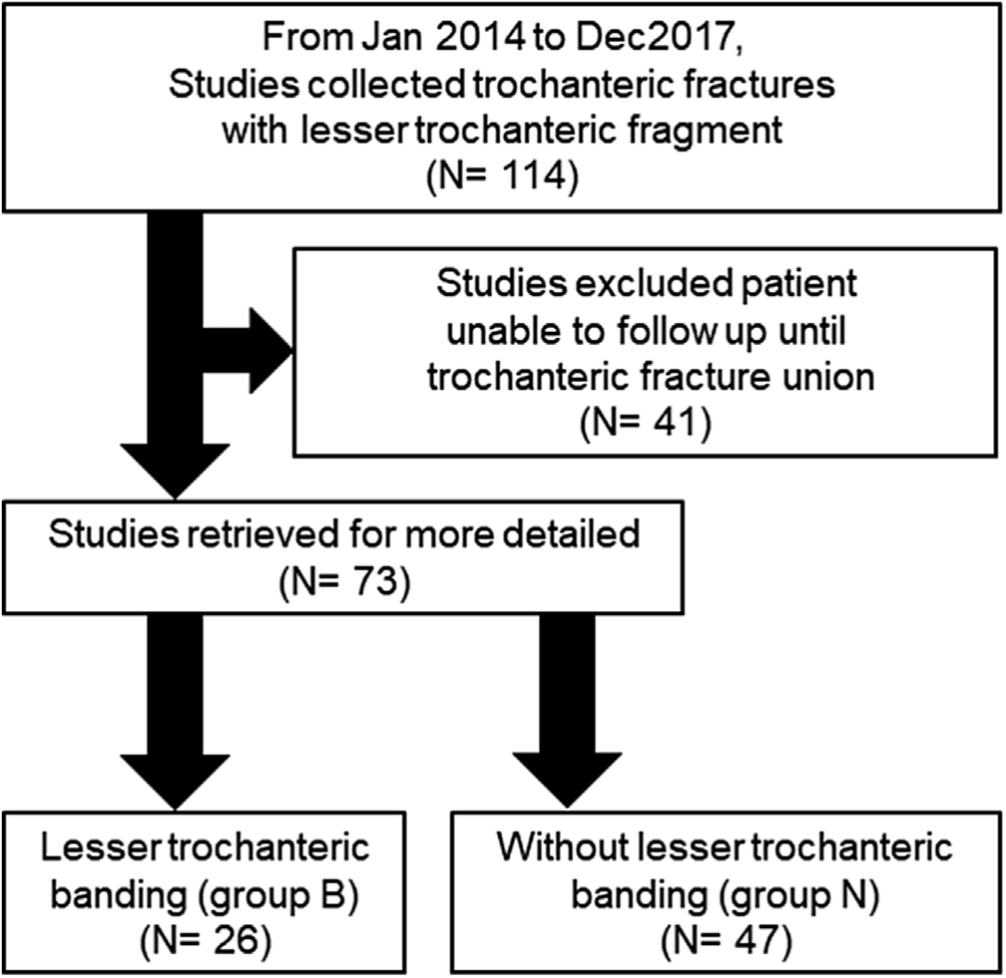
Flow diagram that describes the number of studies.

From January 2014 to December 2017, we retrospectively reviewed the medical records of 114 trochanteric fracture patients with lesser trochanter fragments treated using proximal intramedullary nail at a single trauma center (Kainan Hospital, Yatomi, Japan). Informed consent was obtained from all patients enrolled in this study. The inclusion criteria included unstable trochanteric fractures with displaced lesser trochanter fragment (AO/OTA 31-A2 and 31-A3.3 [11]) treated using Gamma Nail^®^ (Stryker Co., Ltd., USA), INTERTAN^®^ (Smith and Nephew Co., Ltd., UK), TFNA^®^ (DePuy Synthes Co., Ltd., USA), AFFIXUS^®^ (Zimmer Biomet Co., Ltd., USA), Unicorn^®^ (HOYA Co., Ltd., Japan), and CTC Nail^®^ (KISCO Co., Ltd., Japan). Patients who were followed up until the achievement of trochanteric fracture union were included in this study. The exclusion criteria were patients with subtrochanteric, pathological, and open fractures as well as those with a follow-up period of <12 months. According to the criteria, 41 patients were excluded and 73 patients were included in this study. From 2014 to 2015, lesser trochanter fragment banding was not performed; however, from 2016 to 2017, it was performed according to the following indications: one band was used for the trochanteric fracture with lesser trochanter fragment, with 1–2 cm length of the distal cortex from the lesser trochanteric base, and two bands were used for the fracture with >2 cm length of the distal cortex from the base. Using these indications, 73 cases were divided into the lesser trochanter banding group (group B, *n* = 26) and the no banding group (group N, *n* = 47) (Figure 1).

### Surgical procedure

**Figure 2:**
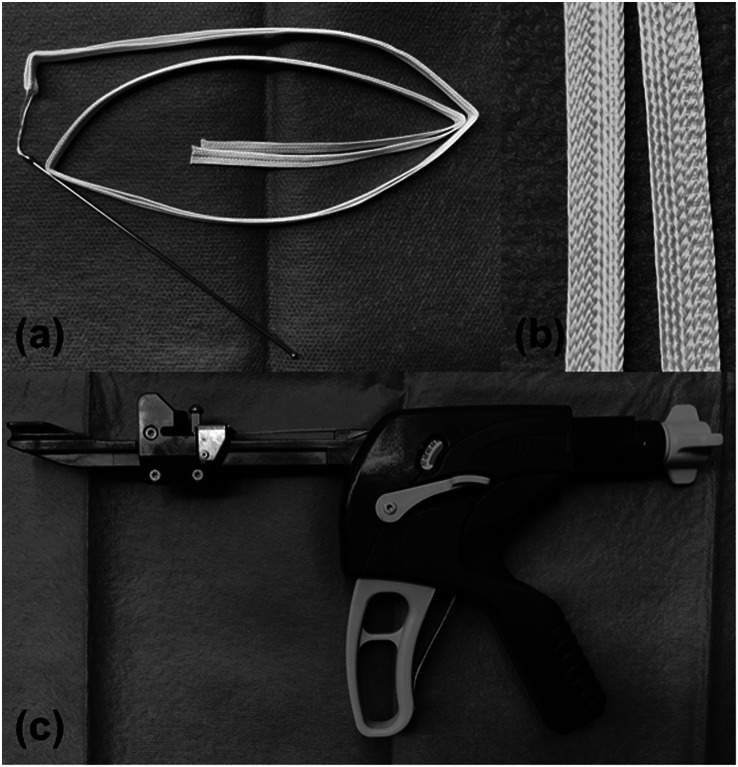
These instruments were used in our study. (a) A picture of the UHMWPE tape, Nesplon tape^®^. (b) A close-up picture of the tape. The Nesplon tape^®^ is a braided tape of 5-mm width that possesses high tensile strength and flexibility. (c) A dedicated tensioner device, called tightening gun^®^, is able to apply an initial strength of 200 N to the tape when making a knot.

**Figure 3:**
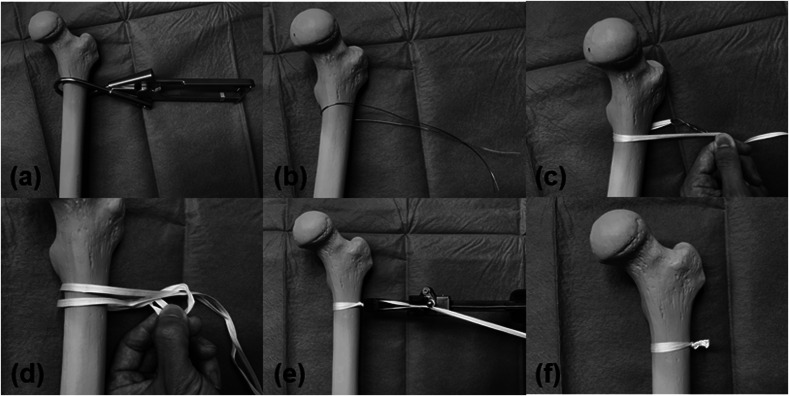
These pictures are the summaries of our surgical technique. (a, b) Cerclage soft steel wire over the lesser trochanter. We recommend using a cerclage passer^®^ for easy cerclage. (c) The Nesplon tape^®^ was passed using soft steel wire. (d, e, f) Strong knots were made using a tightening gun, and the tape was cut at the distal end.

All fractures were treated on a traction operative table under general or spinal anesthesia. Three skin incisions for the nail, lag screw, and distal locking screw insertion were made on the lateral aspect of the femur. Once the fracture was reduced, the intramedullary nail, lag screw, and distal screw were inserted using an image intensifier. The short, medium, or long nail was selected depending on the decision of the senior orthopedic trauma surgeon. After fixing the trochanteric fracture, a soft stainless-steel wire with a 1.2-mm diameter was passed over the lesser trochanter and femoral shaft. To avoid damaging arteries around the femur, the wire must be inserted close to the bone surface. Cerclage Passer^®^ (DePuy Synthes, USA) was used to gently insert the wire into the soft tissues with ease. The level of wiring was within 1 cm below the distal base of the lesser trochanter. Appropriate positioning of the wire was confirmed using an image intensifier. The soft stainless-steel wire was subsequently replaced with a Nesplon tape^®^ (Alfresa Pharma Co., Ltd., Japan), which is an ultrahigh-molecular-weight polyethylene (UHMWPE) nonabsorbable tape with 5-mm width (Figures 2a and 2b). The replacement was performed by inserting the looped Nesplon tape® into a twisted loop at one end of the wire and pulling the other end. The lesser trochanter fragment was tightened using the double-loop sliding knot technique as previously described [14] and then made a strong knot with a tensioning device (Alfresa Pharma Co., Ltd., Japan (Figure 2c). Finally, the surgery was completed after cutting the end of the knot and making a layer suture. All patients were allowed full weight-bearing on the day after the surgery. For better clarity, we have summarized this banding procedure in Figure 3.

### Radiographic assessment

**Figure 4:**
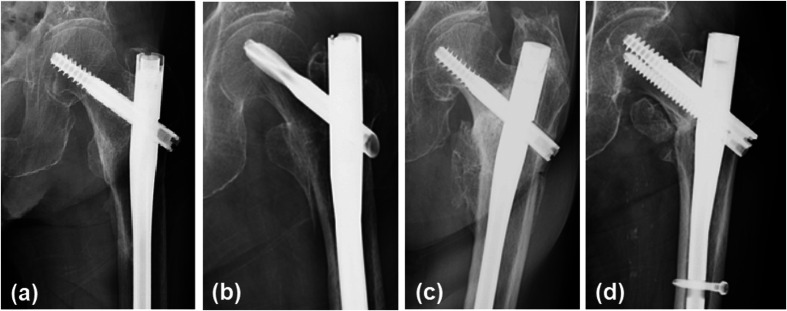
These radiographs show the deformity degree. (a) The radiograph presented mild deformity but showed complete bone union. Slight ossification was observed around the tape. (b) The radiograph presented moderate deformity but also showed complete bone union with 5–10 mm displacement of the lesser trochanter fragment. (c) The radiograph presented severe deformity with the displacement of lesser trochanter fragment to >1 cm from the base. (d) The radiograph showed ununited fragment. The displaced fragment shifted from the original position and was observed in the anterior proximal aspect of the femur. This may be affected by the iliopsoas muscle force.

The radiographs and/or computed tomography images at 3 months after the surgery were assessed to evaluate bone union of the lesser trochanter fragments. Bone union was defined as the existence of bridging callus formation between the lesser trochanter fragment and femoral shaft. The deformity degree of the lesser trochanteric fracture was also assessed, which was defined as follows: mild deformity, <5 mm deformity fusion; moderate deformity, 5–10 mm; severe deformity, >10 mm; and nonunion (Figure 4).

### Statistical analysis

Comparison of categorical and continuous variables between the two groups was evaluated using Fisher’s exact test and Mann–Whitney *U* test, respectively. The effect of banding treatment on the primary outcome was evaluated using the odds ratio. *P* < 0.05 was considered statistically significant. The R statistics package version 3.5.1 (R Core Team, Foundation for Statistical Computing, Vienna, Austria) was used for all analyses.

## Results

The average age was 83.5 (79.3–85.8) years in group B and 84.0 (81.0–89.5) years in group N (*P* = 0.659). The average follow-up period was 21.5 (13.3–37.9) months in group B and 18.8 (13.6–31.4) months in group N (*P* = 0.950). No significant difference was observed in sex (*P* = 0.410), operation side (*P* = 0.626), and AO classification (*P* = 0.314) between these groups (Table 1). Regarding the nail length, we found that group N tended to use short nails and group B tended to use long nails (*P* < 0.001). In group B, 19 patients were treated with one band and 7 were treated with two bands.

**Table 1:** Background of patients.

	Group B (*n* = 26)	Group N (*n* = 47)	*P* value
Mean age (years, range)	83.5 (79.3–85.8)	84.0 (81.0–89.5)	0.659
Follow-up period (months, range)	21.5 (13.3–37.9)	18.8 (13.6–31.4)	0.950
Sex			0.410
Male	5	14	
Female	21	33	
Operation side			0.626
Right	13	20	
Left	13	27	
AO classification			0.314
31-A2	21	42	
31-A3.3	5	5	
Length of nail			<0.001
Short	6	32	
Middle	4	3	
Long	16	12	
Number of banding			
One	19	0	
Two	7	0	
Bone union	24	30	0.011
Deformity			
Mild deformity	11	2	<0.001
Moderate deformity (<10 mm)	9	4	<0.001
Severe deformity (≧10 mm)	4	24	<0.001
Nonunion	2	17	<0.001

Bone union of the lesser trochanter fragment was achieved in 24 patients (92%) in group B and 30 (64%) in group N (*P* = 0.011). In group B, 11 patients exhibited mild deformity, 9 exhibited moderate deformity, and 4 exhibited severe deformity. In group N, 24 patients exhibited severe deformity, 4 exhibited moderate deformity, and 2 exhibited mild deformity. During the follow-up period, 2 patients in group B and 17 in group N showed nonunion. Compared with group N, group B showed a significantly increased number of mild and moderate deformities but decreased number of severe deformities and nonunion (*P* < 0.001). Postoperative complications, such as vascular damage, avascular necrosis of the femoral head, and deep infection, were not observed in both groups.

## Discussion

In the present study, we evaluated the efficacy of banding with the lesser trochanter fragment using nonabsorbable tape for the treatment of trochanteric fractures and found that banding increased the ratio of the bone union of the lesser trochanter fragment and decreased the deformity degree. The displacement of lesser trochanter fragments is associated with a poor clinical outcome and postoperative complications such as implant failure, nonunion, loss of reduction, and severe thigh pain [12]. The iliopsoas muscle is known to be involved in these issues. The iliopsoas muscle, which arises from the iliac fossa of the pelvis and lumbar vertebra and attaches to the lesser trochanter, regulates hip joint flexion and rotation force. In contrast, medial cortex comminution, including lesser trochanter fragments caused by trochanteric fractures, leads to proximal femur instability [15]. Ehrnthaller et al. reported that lesser trochanter fragment fixation with wiring technique increased the stiffness of femur after intramedullary nailing for femoral trochanteric fractures [16]. Thus, the lesser trochanter has a pivotal role in hip joint function and medial cortex support. To reduce displacement of the lesser trochanter fragment, we established a new surgical procedure of banding with the lesser trochanter fragment using nonabsorbable tape in trochanteric fractures. The anatomical study of lesser trochanter and iliopsoas tendon indicated that the iliopsoas tendon generally comprised two or three tendons in several cases [17]. In addition, the footprint of the iliopsoas tendon is located on the anteromedial aspect of the lesser trochanter, with a width of approximately 1.7–2.1 cm [17, 18]. Thus, we passed the nonabsorbable tape around the femur within 1 cm distal to the lesser trochanteric base. In this study, we found that a high union ratio was observed in the banding group and reduction loss was not observed. Our study also showed that the bands were not likely to be torn until the union of the fragments because the displacement of lesser trochanter fragments was not observed after the surgery in the banding group. Considering these findings, it is likely that our surgical procedure of banding with a nonabsorbable tape is sufficiently strong for fixing the lesser trochanter fragment in trochanteric fractures.

**Figure 5:**
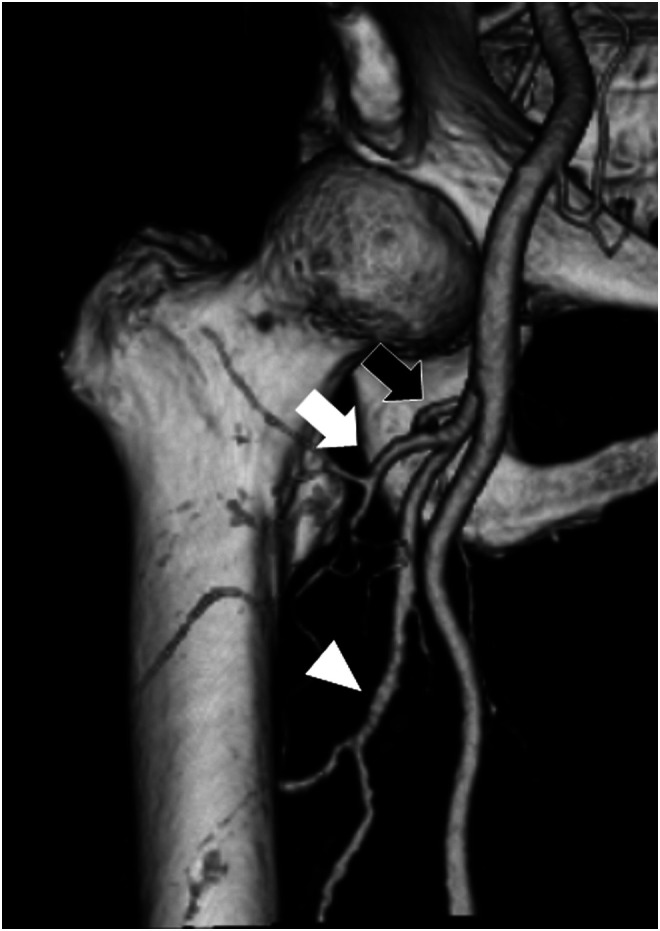
Three-dimensional reconstructed computed tomography image showing arteries that supplied blood to the proximal femur. Lateral femoral circumflex artery (white arrow) runs in front of the femur. MFCA (black arrow) delivered blood to the femoral neck and the head from the posterior aspect of the femur. DFA (white triangle) runs along the femoral shaft.

When using this surgical procedure of banding, all surgeons should monitor femoral blood supply to prevent postoperative complications (Figure 5). The major arteries, such as common femoral artery (CFA), deep femoral artery (DFA), superficial femoral artery, medial femoral circumflex artery (MFCA), lateral femoral circumflex artery, and perforating branches of DFA, run close to the proximal femur [19, 20]. Among these, the MFCA is a vital artery that mainly supplies blood to the femoral head. Thus, damage to the MFCA should be carefully prevented [19, 21]. In their systematic review, Barquet et al. reported that avascular necrosis of the femoral head, which is known as a postoperative complication of trochanteric fractures, occurred after trochanteric fractures in a range between 0.13% and 2.46% and that MFCA is the most important artery for the pathogenesis of avascular necrosis of the femoral head [22]. Recent studies have reported that the MFCA originates from the DFA in 65% and the CFA in 31–32% of cases [23, 24]. In contrast, pseudoaneurysm of the DFA after the surgery is also reported [25, 26]. Although pseudoaneurysm was caused by the fracture itself in some cases, operative fault such as guidewire migration was considered to be the main reason. In our surgical procedure, we used cerclage passer and soft polyethylene tape to prevent damaging soft tissues around the femur. In addition, we avoided banding proximal of the lesser trochanteric tip because the MFCA normally branches off more proximally than the lesser trochanteric aspect. Considering this point, we did not observe any postoperative vascular complications, including avascular necrosis of the femoral head and pseudoaneurysm around the proximal femur. Therefore, our surgical procedure may be a safe method for proximal femoral fractures. Kim et al. introduced the *modified candy package technique* using steel wires to hold the displaced lesser trochanter fragment [13]. Although they showed good clinical results without complications, we are concerned regarding damage to blood vessels around the femur because of steel wire migration. Our procedure is advantageous compared with the method using stemless wires in that the use of nonabsorbable tape does not leave any metallic instruments around the femur. Regarding periosteal blood supply of peritrochanteric fractures and healing rates, it has been reported that cerclage wiring on the femur does not disrupt endosteal blood supply in the cadaveric studies [27]. In this study, all patients showed callus formation around the banding site and bone atrophy was not observed in the proximal femur after the surgery. Thus, it is likely that our surgical procedure may not affect the periosteal blood supply of peritrochanteric fractures and healing rates.

The Nesplon tape^®^ is a UHMWPE fiber tape with properties of high strength and flexibility [28, 29]. UHMWPE tapes have already been employed in several surgical fields such as spinal fusion and hip arthroplasty [30, 31]. We used a dedicated tensioner device (Alfresa Pharma Co., Ltd., Osaka, Japan) to apply the initial tension of 200 N to the fiber tape (Figure 2). The UHMWPE tape has been reported to have a higher cut-through strength than steel wires or cables [29]. In this study, almost all lesser trochanter fragments were well reduced and not displaced until bone union after lesser trochanter fragment banding, whereas most fragments were displaced within 2 weeks after the surgery without banding. However, fixing the lesser trochanter fragment when the fragments are comminuted is difficult. Checking the wiring position by an image intensifier is indispensable to confirm the band position during the surgery.

There are several limitations of this study. First, because the sample size is small, increasing the number of cases to assure stronger evidence is necessary. Second, because patients who could not be followed up were excluded from the analysis, selection bias may have occurred. However, it is speculated that many cases of follow-up failure were accidental, such as death and being hospitalized to another hospital after the surgery. Because it is unlikely that the presence or absence of banding and/or the results would be the cause of the inability to follow-up, we consider that the effect of selection bias is modest in this study. Third, there is a facility bias in this study. Our data were obtained from a single trauma center. The data from other facilities will be needed to ensure external validity. Finally, because this study is a retrospective observational study, a randomized prospective study will be needed in the future.

## Conclusions

Our surgical procedure using the Nesplon tape^®^ provided a high union ratio of the lesser trochanter fragment without any complications. The banding of lesser trochanter fragment in trochanteric fractures could be an effective technique to reconstruct the anatomical iliopsoas-lesser trochanter mechanism.

## Authors’ contributions

T.U. and N.T. designed the study. T.U. and N.T. performed the surgery. T.U. and K.N. analyzed the data. T.U., N.T, and G.K. drafted the manuscript. N.T., I.S., Y.U., and H.M. critically reviewed the manuscript. All authors have read and approved the final manuscript before submission.

## Conflicts of interest

The authors declare that they have no conflict of interest.

## Grant

The present study was supported in part by a Grant-in-Aid for Scientific Research (19K18471) from the Ministry of Education, Culture, Sports, Science and Technology of Japan.
